# Effect of Australian Propolis from Stingless Bees (*Tetragonula carbonaria*) on Pre-Contracted Human and Porcine Isolated Arteries

**DOI:** 10.1371/journal.pone.0081297

**Published:** 2013-11-15

**Authors:** Flavia C. Massaro, Peter R. Brooks, Helen M. Wallace, Vianne Nsengiyumva, Lorraine Narokai, Fraser D. Russell

**Affiliations:** Faculty of Science, Health, Education and Engineering, University of the Sunshine Coast, Maroochydore, Queensland, Australia; University of Padua, Italy

## Abstract

Bee propolis is a mixture of plant resins and bee secretions. While bioactivity of honeybee propolis has been reported previously, information is limited on propolis from Australian stingless bees (*Tetragonula carbonaria*). The aim of this study was to investigate possible vasomodulatory effects of propolis in KCl-precontracted porcine coronary arteries using an *ex vivo* tissue bath assay. Polar extracts of propolis produced a dose-dependent relaxant response (EC_50_=44.7±7.0 μg/ml), which was unaffected by endothelial denudation, suggesting a direct effect on smooth muscle. Propolis markedly attenuated a contractile response to Ca^2+^ in vessels that were depolarised with 60 mM KCl, in Ca^2+^-free Krebs solution. Propolis (160 µg/ml) reduced vascular tone in KCl pre-contracted vessels to near-baseline levels over 90 min, and this effect was partially reversible with 6h washout. Some loss in membrane integrity, but no loss in mitochondrial function was detected after 90 min exposure of human cultured umbilical vein endothelial cells to 160 µg/ml propolis. We conclude that Australian stingless bee (*T. carbonaria*) propolis relaxes porcine coronary artery in an endothelial-independent manner that involves inhibition of voltage-gated Ca^2+^ channels. This effect is partially and slowly reversible upon washout. Further studies are required to determine the therapeutic potential of Australian stingless bee propolis for conditions in which vascular supply is compromised.

## Introduction

Bee propolis is a resinous material that bees produce from plant resins and their body secretions [1]. The chemical composition of propolis reflects the botanical sources from which the bees forage [2]. Honeybee (*Apis mellifera*) propolis has been more extensively studied than its stingless bee (tribe Meliponini) counterpart [2]. In a recent study investigating the chemical composition of New Zealand honeybee and Australian stingless bee propolis, we identified compounds that were only present in honeybee propolis (eg. pinocembrin and galangin), or only present in stingless bee propolis (eg. gallic acid and pimaric acid) [3]. Various bioactivities have been ascribed to some of the 300+ compounds present in honeybee propolis, including antioxidant, antimicrobial, antiinflammatory and antitumour effects [4-6]. For example, pretreatment of Raw 264.7 cells with an ethanolic extract of propolis inhibited lipopolysaccharide-stimulated pro-inflammatory signalling pathways including the phosphorylation of inhibitory protein κB, c-Jun NH_2_-terminal kinase 1/2, and p38 mitogen-activated protein kinase [7]. Synergistic activity by compounds within propolis is likely [8,9]. For example, antitumour effects were significantly greater for the water-soluble derivatives than for the combined effect of the individual polyphenolic compounds present within ethanolic extracts of Croatian and Brazilian honeybee propolis [8].

Over the past 5 years, much interest has focussed on the potential use of propolis for the treatment of injury and disease, and this has been investigated mainly using animal models or cell lines. For example propolis and its constituents have been reported to produce benefits in models of hypertension [10], wounds [11,12], burns [13], cancer [14] and septic arthritis [15]. The antihypertensive effects of propolis were investigated in spontaneously hypertensive rat [10], and nitric oxide synthase-inhibited rat models of hypertension [16]. Fractions of an ethanolic extract of propolis from Minas Gerai state in Brazil reduced blood pressure in the spontaneously hypertensive rats, with activity ascribed to the flavonoids dihydrokaempferide, isosakuranetin and betuletol [10]. Propolis was found to cause vasorelaxation [10,17], and inhibit activity of the catecholamine-producing enzyme, tyrosine hydroxylase [16], suggesting possible mechanisms for its blood pressure lowering effects. The effect of propolis in human hypertension is not yet known. In the vasculature, honeybee propolis modulates blood vessel function by stimulating vasorelaxation [10,17], inhibiting angiogenesis [18,19], and protecting against atherosclerotic lesion formation [20], raising hopes for the development of new therapeutics in the treatment of hypertension, cancer and ischaemic heart disease. These studies have all used honeybee propolis in their investigations. We have previously reported on the chemical composition of propolis from one species of Australian stingless bee (*Tetragonula carbonaria*) [3]. We showed in that study that propolis had antioxidant activity and inhibited 5-lipoxygenase in cell-free assays. Our study identified the presence of pimaric acid in propolis [3], whilst another study examining bioactivity of extracts from roots of *Viguiera arenaria* found that pimaric acid relaxes rat thoracic aorta [21]. The bioactivity of Australian stingless bee propolis in cardiovascular preparations is presently unknown, and so the aim of this study was to investigate its potential to modulate vascular tone.

## Materials and Methods

### Materials

A bulk sample of stingless bee propolis was obtained from 40 hives from within suburban areas of South East Queensland. *T. carbonaria* is known to extensively forage on eucalypt resins, in particular *C. torelliana* and this species shapes their chemical ecology [22,23]. After removal of debris and honey using water, the propolis was unified into a single homogeneous sample. New Zealand honeybee (*A. mellifera*) propolis was obtained from ethanolic tinctures, provided by Comvita New Zealand. Samples were stored in sealed plastic containers at 4°C until use. 

Gallic acid, NG-nitro-L-arginine methyl ester (L-NAME), indomethacin, DMSO, ethyl acetate (EtOAc), diethyl ether (Et_2_O), rat tail collagen type I, 3-(4,5-dimethylthiazol-2-yl)-2,5-diphenyltetrazolium bromide (MTT)-based *in vitro* toxicology assay kit, and lactate dehydrogenase-based toxicology assay kit were purchased from Sigma-Aldrich (Castle Hill, NSW, Australia). Pimaric acid reference compound was from Santa Cruz Biotechnology, CA, USA. Medium 199, Hanks’ Balanced Salt Solution (HBSS), fetal calf serum, collagenase Type II, heparin 1A, fungizone antimycotic liquid, Glutamax-I supplement, and penicillin-streptomycin were purchased from Invitrogen Australia (Rowville, VIC, Australia). All other reagents were of analytical grade.

### Preparation of propolis

Five extracts of stingless bee propolis were prepared. Propolis (5% w/v) was extracted by maceration in hexane:methanol (1:1) for 24h, then paper filtered by gravity. A portion of the methanol extract was dissolved in diethyl ether:sodium carbonate (2% saturated) and partitioned into an ether extract and an aqueous extract. The aqueous extract was acidified by 0.1 M hydrochloric acid then extracted using ethyl acetate. The ethanolic extract of stingless bee propolis was obtained by maceration of raw material in 95% ethanol (5% w/v) and the solution was paper-filtered by gravity. Commercial batches of honeybee propolis contained 172 mg/mL of extracts in 62% ethanol. The five liquid extracts were dried using a rotary evaporator at 40°C and the dry polar residues were weighed and reconstituted to 61 mg/mL using 99% DMSO. These stock solutions were used at the final concentrations indicated for the bioassays. All samples of propolis used in this study were chromatographed prior to the bioassays to confirm their chemical composition. Chemical composition was determined using gas chromatography-mass spectroscopy (GC-MS), as previously described [3].

### Tissue bath experiments using porcine isolated coronary artery and human isolated umbilical artery

Pig hearts were collected from a local abattoir and placed immediately in Krebs’ Henseleit buffer (125 mM Na^+^, 5 mM K^+^, 2.25 mM Ca^2+^, 0.5 mM Mg^2+^, 98.5 mM Cl^-^, 0.5 mM SO_4_^2-^, 32 mM HCO_3_^-^, 1 mM HPO_4_^2-^, 0.04 mM EDTA, 11.7 mM glucose; pH 7.4), pre-bubbled with 5% CO_2_ in oxygen, and transported to the University laboratory on ice. The circumflex coronary artery was dissected free from the heart and cut into ~1.5 cm segments using scissors. Vessels were set up in jacketed tissue baths (37°C) containing 25 ml Krebs’ Henseleit buffer that was bubbled with 5% CO_2_ in oxygen. Vessels were allowed to equilibrate in the baths for 1 h under 40-50 mN resting tension. During the equilibration period, vessels were contracted twice with 60 mM KCl followed each time by a washout with Krebs’ Henseleit solution. Vessels were pre-contracted with 60 mM KCl and then exposed to a single concentration of propolis (160 µg/ml), to increasing concentrations of propolis (0.8-160 µg/ml), or to isovolumetric solvent (DMSO; ≤0.26% v/v). Following washout, vessels were contracted with 60 mM KCl, either as a single administration or as repeat administrations with half-hourly washouts over 6 h. Some vessels were pre-contracted with 2 mM histamine.

The effect of propolis was also investigated on human umbilical arteries. Human umbilical cords were obtained from patients undergoing childbirth via Caesarean section at Nambour General Hospital with approval from the Human Research Ethics Committees of the University of the Sunshine Coast (S/09/221) and Royal Brisbane and Women’s Hospital (HREC/09/QRBW/184), and Site Specific Assessment approval from Nambour General Hospital (SSA/09/QNB/22). Arteries were dissected free at the juncture of the umbilical cord with the placenta. These vessels were pre-contracted with 60 mM KCl and then exposed to 160 µg/ml propolis or isovolumetric DMSO. Developed tension in the blood vessels was recorded using Powerlab 26T with Chart 5 software (AD Instruments Pty Ltd, NSW, Australia) and expressed as a percentage of the initial response to 60 mM KCl. 

 The role of the endothelium in mediating the relaxant response to propolis, some vessels were denuded of endothelial cells by rubbing the lumen with a roughened metallic probe. Successful endothelial denudation was determined by visual inspection of 20 µm transverse sections of vessel cut using a cryostat (Reichert Jung, model 1800) and stained with hematoxylin and eosin (data not shown). Endothelial-intact, and endothelial-denuded vessels were contracted with 60 mM KCl, and then exposed to 40 and 160 μg/ml propolis.

 To examine the role of voltage-gated calcium channels in the relaxant response to propolis, coronary arteries were equilibrated in Krebs solution for 2h, exposing the tissues to 60 mM KCl to ensure tissue viability. Tissues were then equilibrated in Ca^2+^-free Krebs solution containing 0.06 mM EDTA for 2h, with six replicate sequences involving washout followed by addition of 60 mM KCl. The final exposure of tissue to KCl produced a negligible contractile response. Tissues were then treated with either 160 μg/ml propolis or DMSO (0.26%) for 30 min in the presence of 60 mM KCl. Without washout, a concentration-effect curve was constructed to Ca^2+^ (32 μM-10 mM), before adding the Ca^2+^ channel inhibitor, nifedipine (10 μM) to the bath.

### Cell viability assays using human cultured umbilical vein endothelial cells (HUVECs)

Umbilical cords were collected as described above. The umbilical vein was flushed with Dulbecco’s phosphate buffered saline to remove blood, and then administered with 1 mg/mL collagenase type II solution (15-20 min, 22°C). The collagenase solution was recovered, spun (400xg, 5 min, 22°C), and the pellet was resuspended in M199 media containing 20% fetal calf serum, 50 μg/mL penicillin/streptomycin, 2.5 μg/mL fungizone and 2 mM Glutamax-I (20% media). Cells were seeded onto 12-well collagen-coated plates or collagen-coated 13 mm coverslips and grown to confluence in a 5% CO_2_ incubator at 37°C. Cells were incubated in the absence or presence of 20-160 µg/ml propolis, or to isovolumetric solvent (0.26% DMSO) for 90 min at 37°C. Wells were rinsed with HBSS and then exposed to HBSS containing 10% thiazolyl blue tetrazolium bromide for 2h at 37°C. Developed formazan crystals in the 12-well plates were solubilised using MTT Solubilising Solution (Sigma-Aldrich) and absorbance was measured using a spectrophotometer (Versa Max, Associates of Cape Cod Int. Inc., Liverpool, UK) at 570 nm. Background readings measured at 690 nm were subtracted from all values, and cell viability was reported as a percentage of the media control. Coverslips were mounted onto glass microscope slides and formazan crystals in cells were photographed using a brightfield microscope (Olympus CX31) with camera attachment (Olympus Altra 20 Soft Imaging System).

 Cytosolic and total lactate dehydrogenase (LDH) activity was measured in the supernatant collected from intact and solubilised cultured HUVECs using an *in vitro* LDH activity assay kit. Briefly, cells were grown to confluence in 12 well culture plates using 20% M199 media. Media was replaced with Dulbecco’s phosphate buffer solution and cells were incubated with propolis (30-160 µg/ml), or isovolumetric solvents (DMSO or water) for the 160 µg/ml sample of propolis, for 90 min at 37°C. Some wells were treated with lysis buffer (1:10 v/v) for 45 min at 37°C to solubilise membranes and allow determination of total LDH activity. Supernatants were collected from the wells, and spun at 250xg for 4 min to remove cellular debris. Samples were incubated with substrate in an amount equal to twice the volume of medium, for 20 min at 22°C in the dark. Absorbance was read at 490 nm, with subtraction of non-specific absorbance measured at 690 nm. LDH leakage was reported as LDH activity in the supernatant, expressed as a percentage of total cellular LDH activity.

### Data analysis

Propolis constituents were quantitated using the Internal Standard method, as previously described [3]. Concentration-effect curves were plotted using the Levenberg-Marquardt fitting routine based on reduction of chi-square (Microcal Origin Professional V7.5 SR6) and Equation 1.

Equation 1:

Vasodilation (%) =[100 - ((Ytest/YKCl)x100)]%

where Ytest is the response to the sample and YKCl is the maximal response to 60 mM KCl.

Cell viability for cultured HUVECs was calculated using Equation 2.

Equation 2:

Viability (%) = ((Atrig/ADMSO)x100)%

where Atrig is the absorbance of cell lysate from cells treated with *T. carbonaria* propolis and ADMSO is the absorbance of cell lysate from cells treated with DMSO.

 All values shown in the text and figures are mean±SEM. Data was compared using Students *t*- test or one-way ANOVA with Tukey’s post-hoc test using Windows Prism v. 5.02 and Microsoft Excel 2000 v. 9.0.2812. 

## Results

### Chemical composition of bee propolis preparations

The chemical composition of propolis samples was examined using GC-MS analysis. *T. carbonaria* propolis contained several isomers of pimaric acid in both extracts and all subfractions (Table 1). Gallic acid was present in the methanol and ethanol extracts, and only in the ethyl acetate fraction (data not shown). Reference compounds of gallic and pimaric acid conclusively identified these molecules as constitutents of *T. carbonaria* propolis. The main constituents of honeybee propolis from New Zealand tinctures were caffeic acid derivatives and flavonoids (22.3% w/w), while pimaric and gallic acids were not detected.

**Table 1:** Chemical composition of *T. carbonaria* propolis extracts in methanol (MeOH), 96% ethanol (EtOH); fractions in ethyl acetate (EtOAc) and diethyl ether (Et_2_O).

Compound	MeOH	EtOH	EtOAc	Et_2_O
Pimaric acid isomer (#18)	0.5±0.0 %	0.3±0.1 %	0.2±0.0 %	0.9±0.1 %
Pimaric acid isomer (#19)	0.3±0.0 %	0.2±0.1 %	0.1±0.1 %	0.9±0.4 %
Pimaric acid (#20)	5.2±0.7 %	3.8±1.4 %	2.0±0.3 %	9.9±0.3 %
Pimaric acid isomer (#29)	8.4±0.6 %	4.5±0.7 %	8.1±5.0 %	1.8±0.2 %
Total pimaranes	14.4±1.2 %	8.8±2.0 %	10.3±5.2 %	13.4±1.0 %

Pimaric acid (Compound #20) was conclusively identified against a commercially available reference. An internal standard method was used for quantitation of constituents from two independent analyses by GC-MS. Values are mean ±SEM.

### Effects of bee propolis on vascular tone

**Figure 1:**
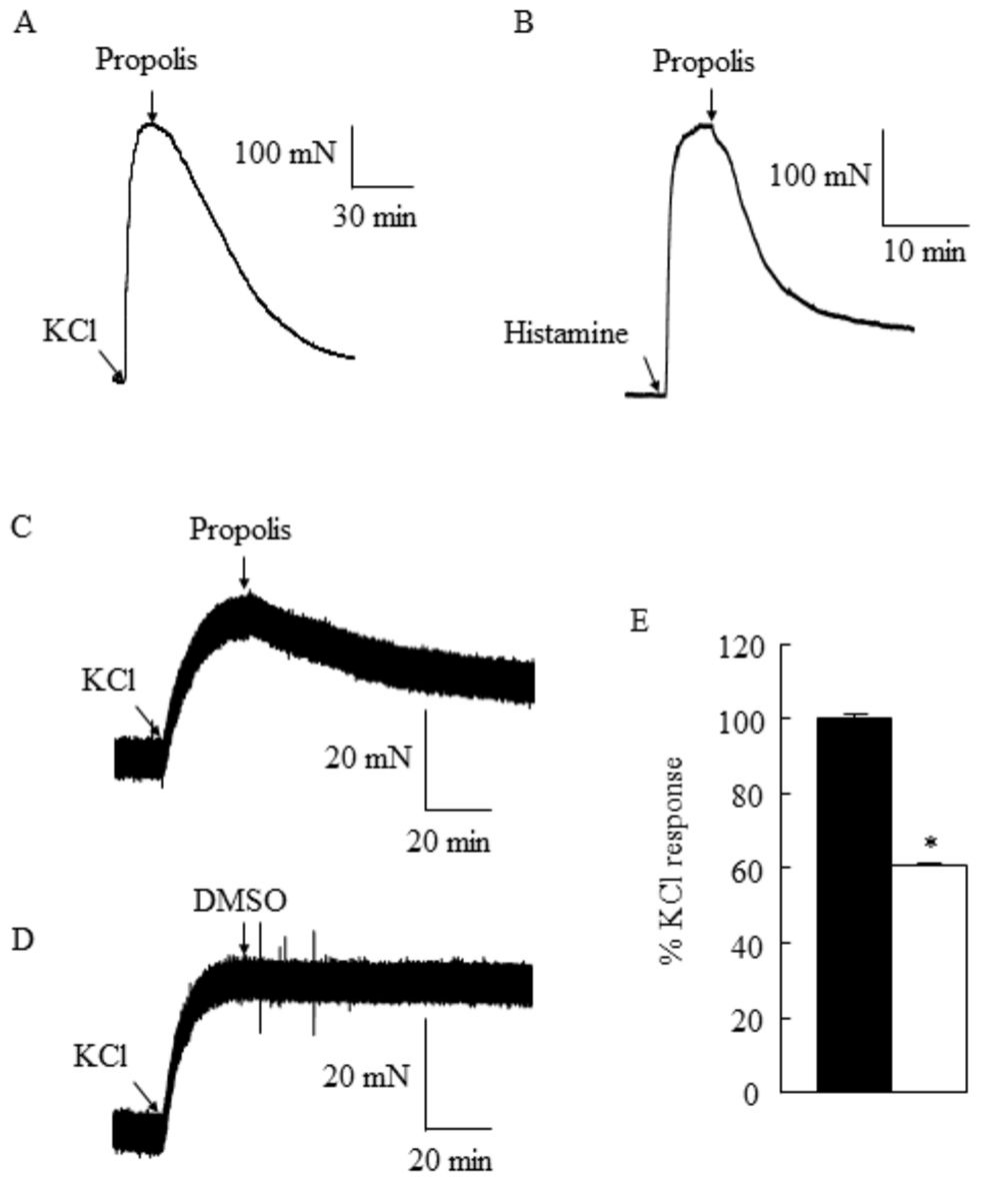
Original traces showing the effect of Australian stingless bee *T. carbonaria* propolis on vascular tone in isolated blood vessel preparations. Propolis relaxed porcine coronary arteries that were pre-contracted with 60 mM KCl (A), or 2 mM histamine (B). Propolis relaxed human umbilical arteries that were pre-contracted with 60 mM KCl (C, E; open bar), while the solvent dimethyl sulfoxide (DMSO) was without effect (D, E; closed bar, n=3, * P<0.05).

Baseline vascular tone, and developed tone in vessels pre-contracted with 60 mM KCl were 46±2 mN (n=6) and 298±47 mN (n=6), respectively. Addition of a single, high concentration of *T. carbonaria* propolis (160 µg/ml, 90min) to pre-contracted vessels reduced tone to near-baseline values (80±12 mN; n=6; Figure 1A). Propolis (160 µg/ml) also caused a reduction in tone in porcine coronary arteries that were pre-contracted with 2 mM histamine (Figure 1B), and in human umbilical arteries that were pre-contracted with 60 mM KCl (Figures 1C, 1E). Isovolumetric DMSO (0.26%) was without effect (Figures 1D, 1E). The rate of relaxation in pig coronary and human umbilical arteries following exposure of tissues to propolis was slow (~90 min to reach full relaxation).

**Figure 2:**
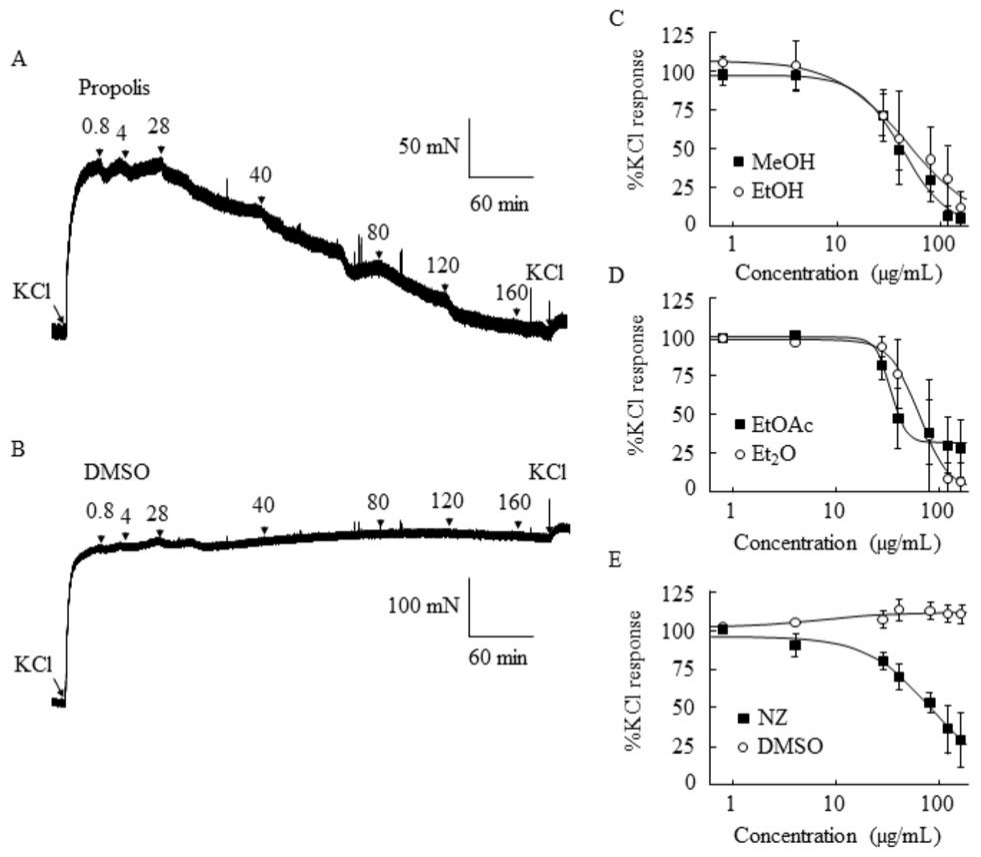
Effect of Australian stingless bee *T. carbonaria* propolis and New Zealand honeybee propolis (NZ) on vascular tone in isolated blood vessel preparations. A methanolic extract of propolis (0.8-160 µg/ml) produced a dose-dependent relaxant response in porcine coronary artery rings (A), while solvent (dimethyl sulfoxide, DMSO) was without effect (B). Concentration effect curves for methanolic (MeOH) and ethanolic (EtOH) extracts of stingless bee propolis (C). Concentration effect curves for ethyl acetate (EtOAc) and diethyl ether (Et_2_O) sub-fractions of the methanolic extract of stingless bee *T. carbonaria* propolis (D). Concentration effect curves for an ethanolic extract of New Zealand honeybee propolis, and DMSO (E).

The methanolic extract of *T. carbonaria* propolis (0.8-160 μg/ml) caused concentration-dependent relaxation of 60 mM KCl-contracted porcine coronary arteries, while isovolumetric solvent (DMSO, 0.001-0.26%) was without effect (Figure 2A, B). The potency of the methanolic extract of *T. carbonaria* propolis (IC_50_, 44.7±7.0 µg/ml; n=5) was similar to that of the ethanolic (IC_50_, 59.8±19.3 μg/ml; n=3), ethyl acetate (IC_50_, 34.6±2.7 μg/ml; n=3), and diethyl ether (IC_50_, 60.9±14.9 μg/ml; n=3) sub-fractions (Figure 2C, D). The potency of *T. carbonaria* propolis preparations was similar to that of the ethanolic tincture of New Zealand honeybee propolis (71.9±17.1 μg/ml; n=3) (Figure 2E). Pimaric acid (1-200 µM) and gallic acid (1-200 µM) reference compounds did not cause a vasorelaxant response (92.7±6.8% and 95.5±4.0% of KCl, respectively), with vascular tone similar to that observed for DMSO (95.9±3.6%; n=3). The addition of 160 µg/ml propolis to the tissue bath did not alter the pH of the Krebs Henseleit solution (pH before and after addition of propolis was 7.6).

**Figure 3:**
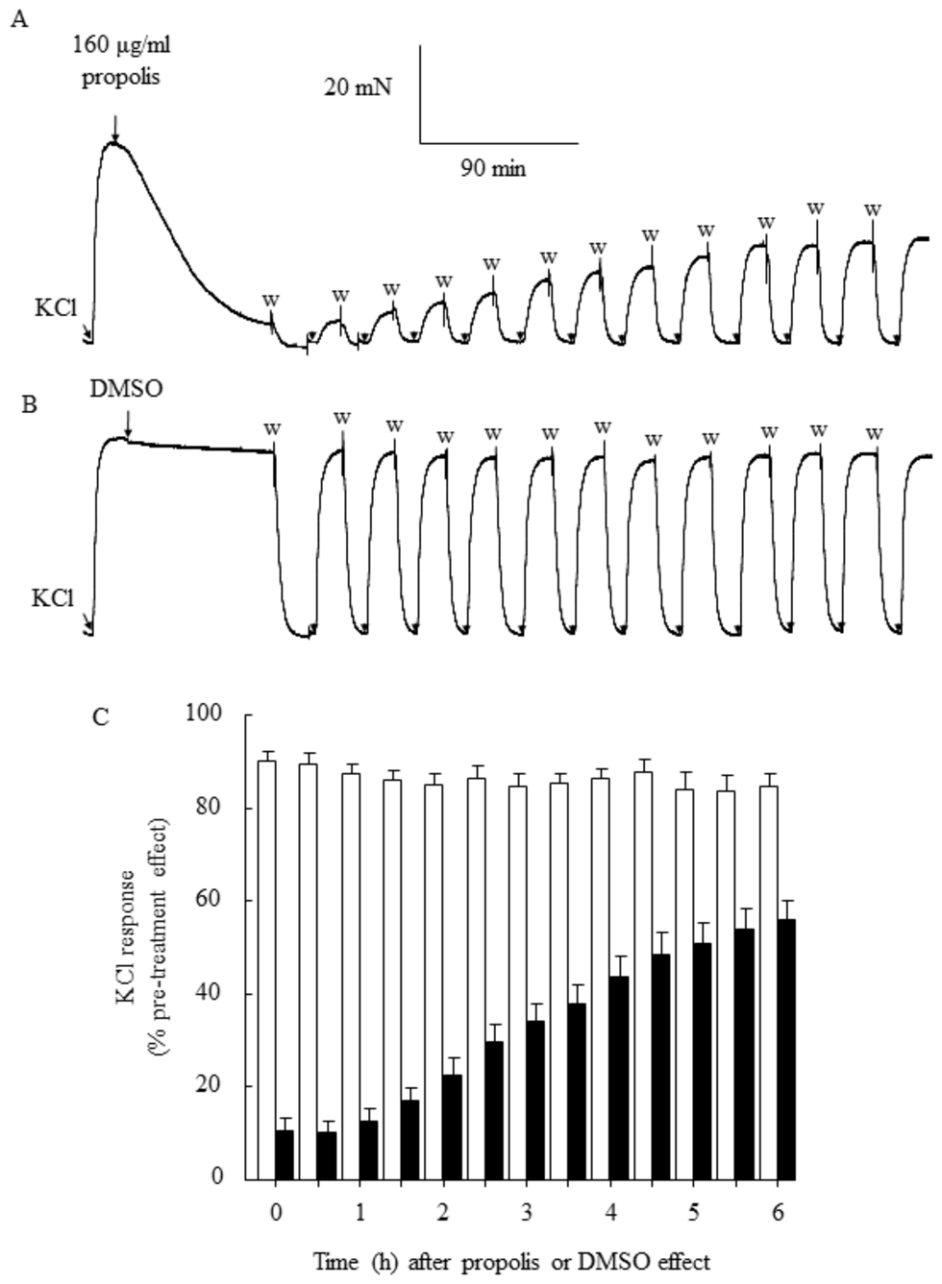
Reversibility of the vasorelaxant response to stingless bee *T. carbonaria* propolis in 60 mM KCl-precontracted porcine coronary arteries. Propolis (160 µg/ml) caused a near-maximal reduction in coronary artery tone within 90 min of incubation. The contractile response to KCl immediately after washout was markedly lower than the KCl response obtained prior to addition of propolis (A). The KCl response was not blunted in dimethyl sulfoxide (DMSO)-treated tissues (B). The relaxant response to propolis was partially reversed during half-hourly wash-KCl cycles over a 6 hour period (open bars, DMSO-treated tissues; closed bars, propolis-treated tissues (C). W, washout.

After washout of propolis, the response to KCl (developed tension, 35±12 mN; n=6) was significantly less than the response to KCl before the addition of propolis (298±47 mN; n=6; P<0.05). The reversibility of relaxant response to 160 µg/ml propolis was therefore examined over a 6h period in which tissues were washed and exposed to 60 mM KCl at half-hourly intervals. The relaxant response was only partially reversible after 6h washout, with maximal response to KCl in propolis-treated tissues (56.02±4.13% of pre-treatment KCl response; n=6) being less than the response to KCl in DMSO-treated, time-control tissues (84.64±2.83% of pre-treatment KCl response; n=3; P<0.05) (Figure 3).

### Effects of bee propolis on cell morphology and viability of HUVECs

**Figure 4:**
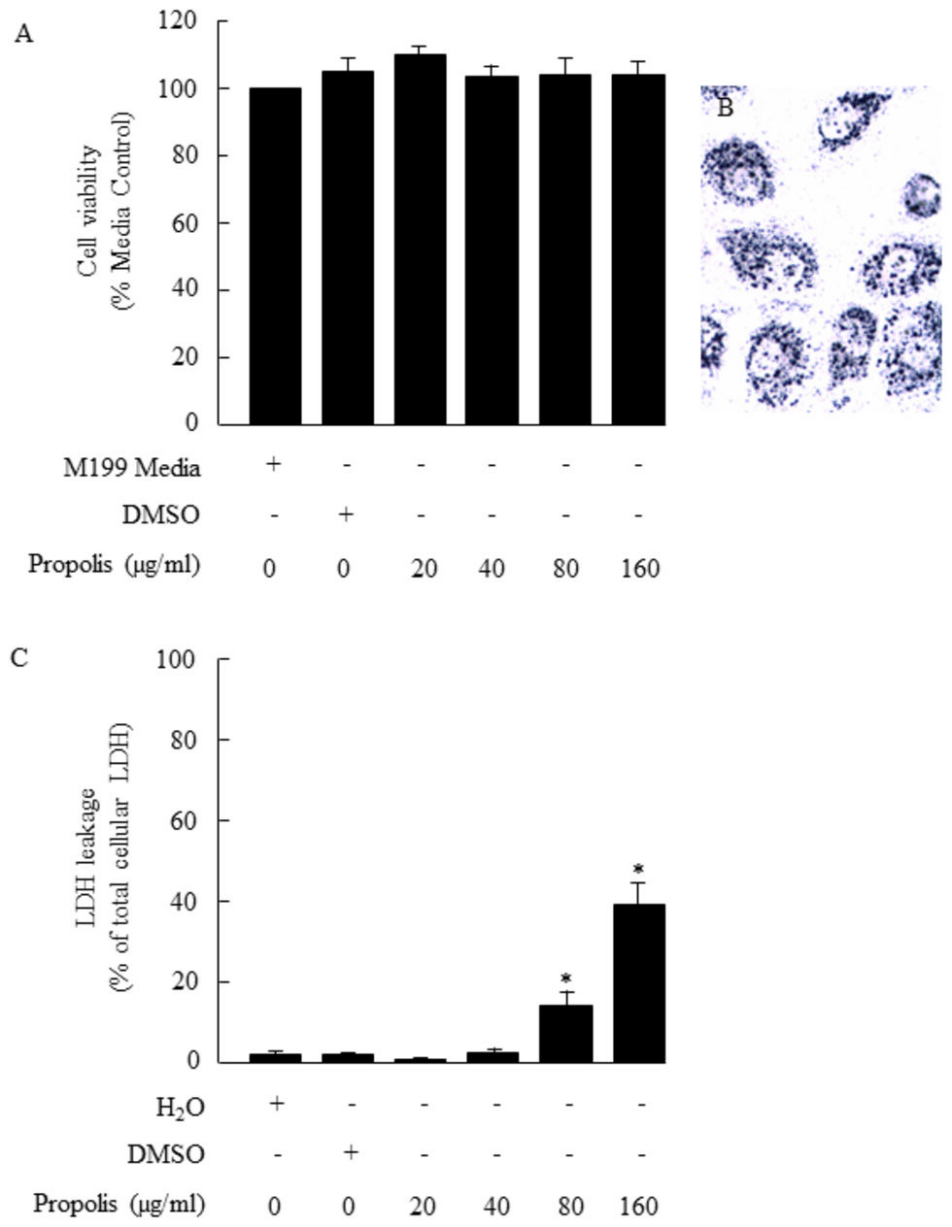
Cell viability assays. MTT assay showing cell viability for human cultured umbilical vein endothelial cells (HUVECs) exposed to stingless bee *T. carbonaria* propolis or dimethyl sulfoxide (DMSO) for 90 min at 37°C (A). Propolis (20-160 µg/ml) and isovolumetric DMSO (0.26%) had no effect on cell viability. A punctuate pattern of formazan crystal staining was evident in the cytoplasm of HUVECs treated with 160 µg/ml propolis for 90 min at 37°C, confirming retention of mitochondrial enzyme activity (B). Propolis (80 and 160 µg/ml) caused some leakage of lactate dehydrogenase (LDH) into the culture medium (C). Water, DMSO (0.26%) and propolis (20 and 40 µg/ml) had no effect on LDH leakage.

We examined the possibility that exposure of cells to *T. carbonaria* propolis might have caused cytotoxicity leading to loss of cell viability. Using cultured HUVECs in an MTT cell toxicity assay, no loss in cell viability was detected when cells were exposed to *T. carbonaria* propolis (Figure 4A). Importantly, similar conditions employed in the tissue bath experiments were used in the cell viability assay (160 µg/ml propolis for 90 min at 37°C). Formazan crystals were visualised in the cytoplasm of the HUVECs using brightfield microscopy (Figure 4B). Using a toxicity assay that was based on lactate dehydrogenase activity, some lactate dehydrogenase activity was detected in the incubation medium (Dulbecco’s phosphate buffer solution) following exposure of cells to 80 and 160 µg/ml propolis (Figure 4C). Lower concentrations (20 and 40 µg/ml) of propolis were without effect. The findings indicate that whilst mitochondrial function appeared to be preserved in cells that were exposed to amounts of propolis up to 160 µg/ml propolis, there was some loss in membrane integrity when propolis was used at ≥80 µg/ml.

**Figure 5:**
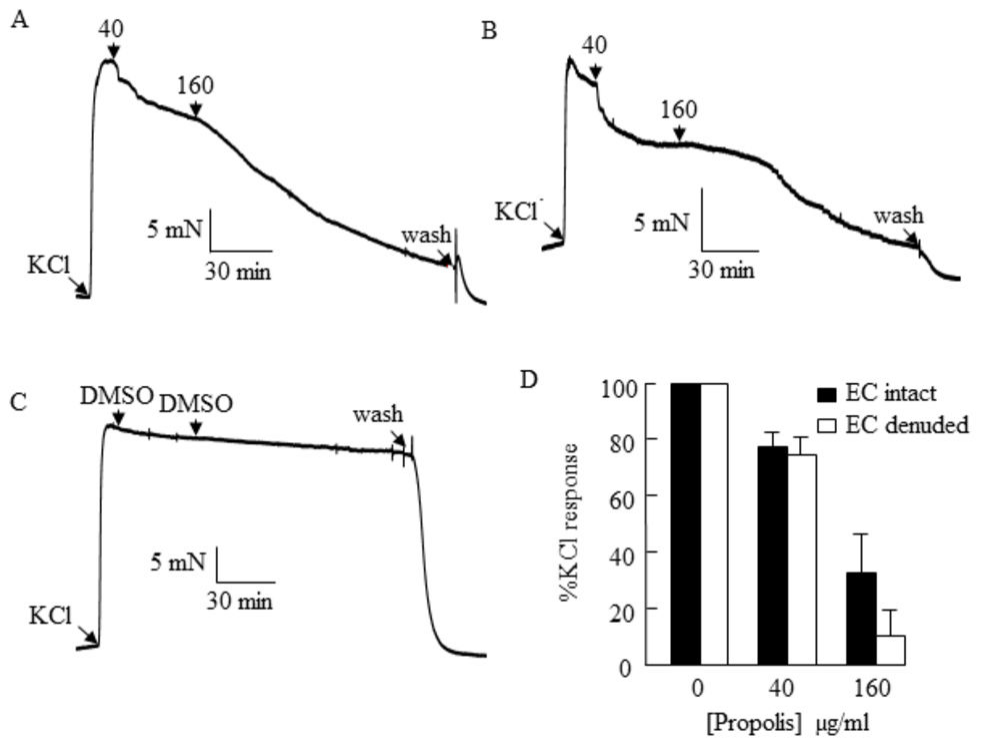
Examination of the role of endothelial cells in the relaxant response to stingless bee *T. carbonaria* propolis in porcine coronary arteries. Propolis (40 and 160 μg/ml) relaxed endothelial-intact (A) and endothelial-denuded coronary arteries (B). No relaxation was observed in endothelial-intact, time-control tissues treated with DMSO (C). There was no difference in relaxant response to propolis in endothelial-intact and endothelial-denuded arteries (D), indicating an endothelial-independent mechanism.

A possible endothelium-dependent mechanism for the vasorelaxant response to *T. carbonaria* propolis (40 and 160 µg/ml) was investigated (Figure 5). The relaxant response to propolis was not attenuated by endothelial cell denudation, suggesting a direct effect of propolis on the underlying smooth muscle cells. Successful removal of the endothelial layer was verified by histological staining of tissue sections taken from the vessels (data not shown).

**Figure 6:**
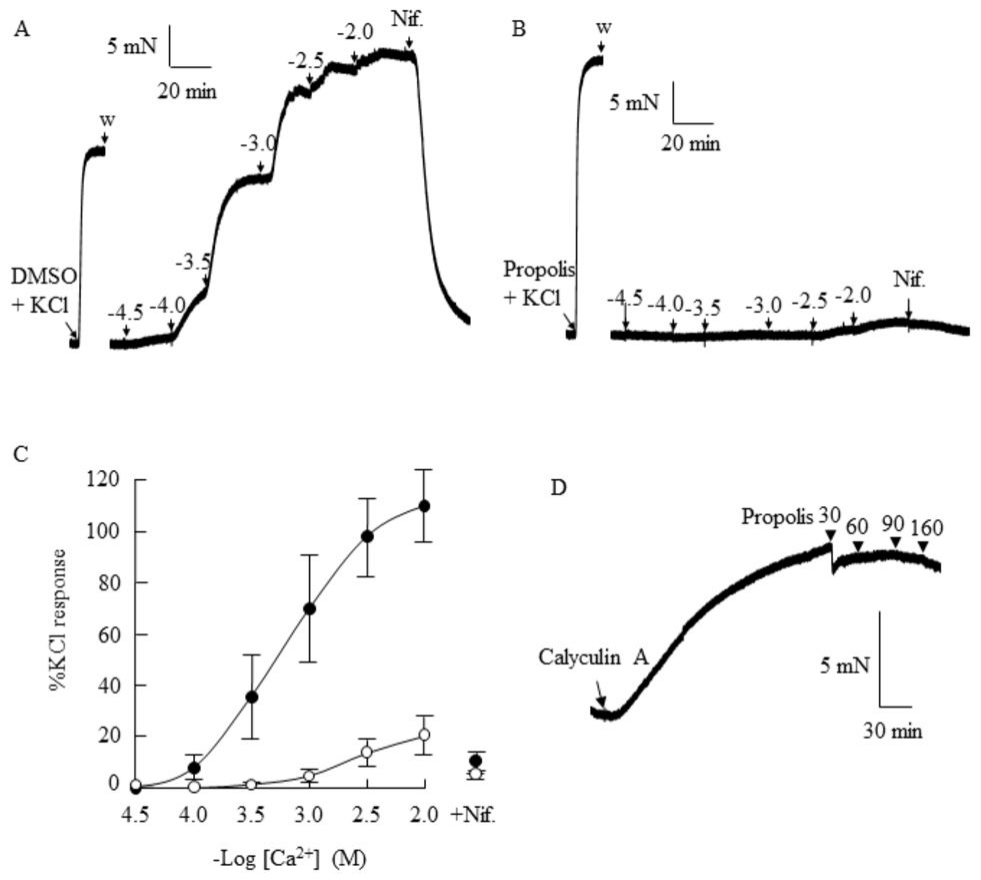
Effect of propolis on voltage-gated calcium channels in endothelial-intact porcine coronary arteries. Arteries were incubated with 60 mM KCl in calcium-free Krebs solution to depolarise the cells (A-C), or with 100 nM calyculin A in Krebs solution (D). In the presence of dimethyl sulfoxide (DMSO), arteries produced a concentration-dependent contractile response to Ca^2+^ (32 μM-10 mM; A, C). The relaxant response was markedly attenuated in the presence of 160 μg/ml propolis (B, C), and responses were blocked by the calcium channel inhibitor, nifedipine (Nif., 10 μM; A-C). Propolis was ineffective in relaxing endothelium-denuded coronary arteries that were pre-contracted with calyculin A (D).

To examine the role of voltage-gated Ca^2+^ channels in the relaxant response to propolis, vessels were incubated in Ca^2+^-free Krebs solution whilst cells were depolarised with 60 mM KCl to open the voltage-gated Ca^2+^ channels. In control tissues incubated with DMSO, Ca^2+^ caused a maximal response that was 109.8±14.0% of the response to KCl (EC_50_ of 0.81±0.19 mM; n=4; Figure 6A-C). The response to Ca^2+^ in the presence of propolis was markedly attenuated (20.4±7.4% of the response to KCl; P<0.05, n=5), with a non-significant trend for a rightward shift in the response curve (EC_50_, 2.3±0.69 mM, n=5). Responses to Ca^2+^ were inhibited by 10 μM nifedipine. Propolis was ineffective in relaxing endothelium-denuded coronary arteries that were pre-contracted with 100 nM calyculin A (Figure 6D).

## Discussion

In this study we investigated the possible modulation of vascular tone by Australian stingless bee (*T. carbonaria*) propolis. We found that propolis caused a vasorelaxant response in vessels that were pre-contracted in a receptor-dependent (histamine) and receptor-independent manner (KCl), and in vessels sourced from different species (human and pig). In porcine coronary arteries, the potency of stingless bee propolis extracts and fractions were similar and in the range, IC_50_, 33-63.7 µg/ml. Chemical analysis revealed several isomeric forms of pimaric acid (Table 1) [3]. We hypothesised that pimaric acid may elicit a vasorelaxant response in porcine coronary artery, similar to that reported previously in rat aorta [21]. However, a reference compound that was identical to Compound #20 had no effect in our blood vessel preparation. Whilst this isomer was not responsible for the vasorelaxant response in the porcine coronary arteries, it comprised only 5.2±0.7% (w/w) of all constituents identified in the methanolic extract, and we therefore do not exclude the possibility that one of the other pimaric acid isomers was active.

The potency of stingless bee propolis preparations was not significantly different to that determined for an ethanolic tincture of New Zealand honeybee propolis in porcine coronary artery (IC_50_, 87.3 µg/ml; this study). Interestingly, the potency of New Zealand propolis determined in our study was lower than that determined for an ethanolic extract of Brazilian green propolis (IC_50_,15.0 µg/ml) and a flavonoid-rich sub-fraction of that ethanolic extract (IC_50_, 4.4 µg/ml) in spontaneously hypertensive rat aorta [10]. Differences may be related to the chemical composition of each sample of propolis (botanical origin), species of animal (pig, rat) and/or tissue type (coronary artery, aorta). 

In this study, the relaxant response to stingless bee propolis was slow to develop, with maximal effect occurring within 90 min. To our knowledge, no other study has examined the kinetics for reversibility of the vasorelaxant response to any type of propolis following washout. After exposure of the coronary artery to stingless bee propolis for 90 min, vascular tone was reduced to near-baseline values. Following initial washout of propolis from the bath, addition of 60 mM KCl produced a contractile effect that was markedly lower than for KCl when applied prior to the addition of propolis. The findings suggest that propolis was either toxic to the tissue, or that the effect of propolis was slowly reversible. Both hypotheses were examined in this study.

Cell viability in the presence of stingless bee propolis was examined using an MTT-based *in vitro* toxicology assay, and a lactate dehydrogenase activity assay, with conditions that approximated those used in the tissue bath experiment (exposure of cells to 160 µg/ml propolis for 90 min at 37°C). Whilst no loss in cell viability was detected using the MTT assay using these conditions, some leakage of lactate dehydrogenase was detected at 80 and 160 µg/ml propolis, suggesting preserved mitochondrial function but some deterioration in membrane integrity. Formazan crystals were visualised in the cytoplasm of the HUVECs, confirming specificity of the MTT assay. The findings with the MTT assay are in contrast to previous reports that showed angiogenesis suppression and chromatic condensation, and disruption of mitochondrial membrane potential in HUVECs by honeybee propolis extracts at <50 μg/ml [24-26]. Differences in chemical composition might contribute to the disparate results. It is noteworthy, however, that experimental conditions were also different: 90 min incubation using fed cells in our study compared to 6-48 h incubation using starved cells in the other studies. Serum starvation is an established stimulus of apoptosis [27,28] and autophagy [29], and it is possible that either of these conditions could have rendered cells more susceptible to the effects of propolis. This hypothesis is supported by a study using pancreatic INS-1(β) cells, which found that fatty acids were not cytotoxic to serum-fed cells, and only induced apoptosis when cells were ‘fragilized’ by serum starvation prior to fatty acid exposure [30]. Importantly in our study, conditions were selected to mimic those used in the tissue bath experiment.

The possibility that the effects of stingless bee propolis were slow to reverse was examined by instigating half-hourly wash – KCl cycles over 6h following attainment of the maximal relaxant response to propolis. The findings confirmed that the effect of propolis was slow to reverse. The response to KCl in propolis-treated tissues increased steadily over the 6h, but remained lower than the response to KCl in DMSO-treated tissues. The findings suggest that a longer washout phase would be required to fully reverse the effects of propolis. However, we cannot exclude the possibility that this propolis chemotype contains a compound (or compounds) that partially and irreversibly relaxes vascular smooth muscle.

We investigated whether an endothelium dependent mechanism might be important in our observed vasodilator response. A previous study showed a nitric oxide-dependent vasorelaxant response to caffeic acid phenethyl ester (CAPE) [17], a constituent previously identified in honeybee propolis. However, in our study, CAPE was not detected in stingless bee propolis. Furthermore, the vasorelaxant response to stingless bee propolis was unaffected by endothelial denudation. Since nitric oxide synthase is expressed in endothelial cells and produces nitric oxide, the findings suggest that stingless bee propolis might mediate its effects by direct interaction with the vascular smooth muscle cells. As the response was observed in vessels that were pre-contracted with a high concentration of KCl (60 mM), mechanisms involving potassium efflux are unlikely [31]. A possible mechanism involves inhibition of voltage-gated calcium channels in the smooth muscle by propolis, similar to that previously reported for NaHS in mouse aorta [32]. To test this hypothesis, we constructed concentration-effect curves to Ca^2+^, in the presence or absence of propolis, in Ca^2+^-free Krebs solution. The findings showed marked attenuation in the contractile response to Ca^2+^ by propolis when cells were depolarized with potassium.

The role of myosin light chain phosphatase in the dephosphorylation of myosin light chain, leading to vascular relaxation is well established [33]. We speculated that if propolis was mediating its vasorelaxant effect through stimulation of myosin light chain phosphatase, it would not be able to reverse a contractile response to a myosin light chain phosphatase inhibitor. We found that propolis was able to relax vessels pre-constricted with histamine and KCl, but was ineffective against calyculin A. Further studies are required to examine this possible mechanism in further detail, including effects on RhoA-dependent signalling pathways. 

In conclusion, this study has identified a marked vasorelaxant response to Australian stingless bee *T. carbonaria* propolis in KCl- and histamine-precontracted porcine isolated coronary arteries. The response was endothelium-independent. We showed that a contractile response to Ca^2+^ in potassium depolarised vessels incubated in Ca^2+^-free Krebs solution was markedly attenuated by propolis. The findings suggest a mechanism involving inhibition of voltage-gated Ca^2+^ channels. Under the experimental conditions used in this study, high concentrations (80 and 160 µg/ml) propolis caused some loss in membrane integrity, but no apparent loss in mitochondrial function. The relaxant response was slowly and partially reversible upon washout. We suggest that Australian stingless bee propolis may have therapeutic potential for management of cardiovascular disorders involving compromised vascular supply, although safety testing is warranted.
